# Supplementary material for: Single-molecule trapping and spectroscopy reveals photophysical heterogeneity of phycobilisomes quenched by Orange Carotenoid Protein
Source: Nat Commun. 2019 Mar 12;10:1172. doi: 10.1038/s41467-019-09084-2 (PMC6414729; doi:10.1038/s41467-019-09084-2)
Supplement: Supplementary file 1 — Supplementary Information [file 41467_2019_9084_MOESM1_ESM.pdf]

SUPPLEMENTARY INFORMATION

Single-molecule trapping and spectroscopy reveals photophysical heterogeneity of phycobilisomes quenched by Orange Carotenoid Protein

Allison H. Squires[1], Peter D. Dahlberg[1], Haijun Liu[2],
Nikki Magdaong[2], Robert E. Blankenship[2], and W.E. Moerner*,[1]

[1] Department of Chemistry, Stanford University, Stanford, California 94305, USA
[2] Departments of Biology and Chemistry, Washington University in St. Louis, St. Louis, Missouri, 63130, USA
* Correspondence and requests for materials should be addressed to W.E.M. (email: wmoerner@stanford.edu)

Table of Contents

Supplementary Notes 3
Supplementary Note 1: Bulk measurements show a low percentage of unquenched complexes in the quenched samples 3
Supplementary Note 2: The electric fields of the ABEL trap do not alter the photophysical properties of analyte molecules........ 4
Supplementary Note 3: Transitions among quenched and unquenched states of the phycobilisome are infrequently observed in the ABEL trap 4
Supplementary Note 4: Alternative combinations of binding sites and non-degenerate binding sites4
Supplementary Figures 6
Supplementary Figure 1: Brightness of unquenched CB-PB is linear with excitation intensity 6
Supplementary Figure 2: Anti-Brownian trapping of single unquenched phycobilisomes under high excitation intensity 7
Supplementary Figure 3: Scatter density plots for unquenched phycobilisome at high excitation power 8
Supplementary Figure 4: (Next page) Extended raw data trace for ABEL trapping of single unquenched phycobilisomes 10
Supplementary Figure 5: Histograms for state U........ 11
Supplementary Figure 6: Histograms for state B 12
Supplementary Figure 7: Histograms for state Q1........ 13
Supplementary Figure 8: Histograms for state Q2........ 14
Supplementary Figure 9: Fluorescence lifetime and brightness of C-PC rods in the ABEL trap..... 15
Supplementary Figure 10: The identity of state B and apparent transitions during trapping 16
Supplementary Figure 11: Compartmental model of OCP-quenched CB-PB phycobilisome for three quenchers at a', d', and e........ 17
Supplementary Tables........ 18
Supplementary Table 1: Connectivity of compartmental model 18
Supplementary Table 2: Compartmental model absorption probabilities........ 19
Supplementary References........ 20

Supplementary Notes

Supplementary Note 1: Bulk measurements show a low percentage of unquenched complexes in the quenched samples

The bulk measurements made in this study (lifetime, emission spectrum) are mutually consistent with a very low percentage of unquenched complexes initially present in the quenched samples (~3.7%). The fluorescence decay of the PB+OCP bulk sample was fit with a bi-exponential decay, which prior to convolution with the IRF and addition of the expected background can be simply written as:

$$P(t) = Ae^{-t/\tau_1} + (1 - A)e^{-t/\tau_2} \quad (1)$$

The fitted parameters for this sample were A = 0.9603, τ_1 = 0.096 ns, and τ_2 = 1.45 ns. By comparing the contributions to the total area under the curve from each lifetime component, we can calculate the proportion of photons under this curve that come from the short lifetime, which we assume to be a quenched state:

$$\%\text{ photons from state Q} = \frac{A\tau_1}{A\tau_1 + (1-A)\tau_2} \times 100\% = 61.5\%\text{ of photons} \quad (2)$$

…and similarly, from the long lifetime, which we assume to be the unquenched state:

$$\%\text{ photons from state U} = \frac{(1-A)\tau_2}{A\tau_1 + (1-A)\tau_2} \times 100\% = 38.5\%\text{ of photons} \quad (3)$$

However, to find the relative number of complexes of each type, we must scale by the relative brightness of each state. The fully quenched complex is 6% of the brightness of an unquenched complex. Therefore:

$$\%\text{ state Q complexes} = \frac{A\tau_1/0.06}{A\tau_1/0.06 + (1-A)\tau_2} \times 100\% = 96.3\%\text{ of complexes} \quad (4)$$

and

$$\%\text{ state U complexes} = \frac{(1-A)\tau_2}{A\tau_1/0.06 + (1-A)\tau_2} \times 100\% = 3.7\%\text{ of complexes} \quad (5)$$

That is, this bulk lifetime decay shows that <5% of complexes are unquenched. We can then calculate the expected total brightness ratio of this quenched sample to an unquenched sample, again assuming that quenched complexes are 6% as bright as unquenched complexes:

$$96.3\% * 0.06 + 3.7\% * 1 = 9.5\% \quad (6)$$

We expect that the bulk sample from the lifetime decay shown in Figure 1e will be about 9.5% as bright as the unquenched sample. This is very close to the ratio of the integrated areas under the spectra shown in Figure 1d, which shows that the quenched sample is 9% as bright as the unquenched sample.

Put another way, we could say that the ratio of emission spectra brightnesses (9%) shown in Figure 1d implies that only about 3% of complexes could be in state U in the quenched sample, if we assume that quenched complexes are 6% as bright as unquenched complexes.

Additional complexities including the presence of some Q_1 complexes and/or the presence of decoupled rods are not included here, and indeed might alter these numbers somewhat. Nevertheless, the close agreement of these calculations to the measurements serves to demonstrate that the bulk fluorescence lifetime data and the bulk fluorescence emission spectrum data are mutually consistent, and indicate that very few unquenched complexes are present in the quenched sample.

Moreover, these numbers also imply that the bulk data are consistent with the reported single-molecule quenching levels (6% for Q_2), as well as with our suggestion that most complexes are quenched into the Q_2 state upon (saturated ratio) binding of OCP.

Supplementary Note 2: The electric fields of the ABEL trap do not alter the photophysical properties of analyte molecules

In the trap, events from quenched complexes are photostable and many seconds in duration. Unquenched complexes (at lower illumination powers) are also photostable. Moreover, the lifetime and emission spectrum of the unquenched complex is identical to bulk. Therefore, it is evident that the ABEL trap does not substantively alter the photophysical properties of the phycobilisome upon trapping. Additionally, the topic of sample perturbation has been addressed in several previous ABEL trap studies – we find that the rotational diffusion,[1] binding and unbinding kinetics,[2] structural conformation of biopolymers,[3] and fluorescence parameters[4-6] are unchanged from their bulk values.

Supplementary Note 3: Transitions among quenched and unquenched states of the phycobilisome are infrequently observed in the ABEL trap

In this work we do not claim to definitively identify transitions among the various states we have identified. This is for three main reasons:

1. Regarding the expectation of observing transitions during a trapping event: Our time-dependent unbinding data (Figure 4) indicates that the PB-OCP complex is relatively stable, and OCP unbinds over the course of hours. Here, we trap each complex for no more than a few seconds. Therefore, we expect that we would only rarely observe transitions during trapping events.
2. We do occasionally observe trapped objects that appear to change their state (a couple of examples of this are evident in the extended raw data set presented in SI Figure S3). However, these events are rare. This is consistent with the expectation described above for a stable PB+OCP complex, but it is also possible that those rare transitions could be replacement events in the ABEL trap, where a second complex (which might be in a different state from the trapped object) enters the trapping area and by chance replaces the initially trapped object. While we work at very low concentration to minimize this possibility, it cannot be discounted in the case of rare events. We therefore do not speculate in the manuscript as to the nature of these events.
3. Moreover, the active observation of binding or unbinding of OCP to the phycobilisome is more relevant to its binding kinetics than to the photophysical states present in the quenched complex, which is the topic of this work.

Supplementary Note 4: Alternative combinations of binding sites and non-degenerate binding sites

In our Discussion, we present simulation results for two OCPs bound at the four pairs of rotationally symmetric compartments. We excluded non-rotationally symmetric combinations and more than two OCPs from consideration because these conditions would generally predict more than two quenched populations except under specific conditions. For example, we expect that three distinct populations would be observed for binding at two asymmetric sites: 1) the doubly-quenched population, 2) one of the single-quenching sites, and 3) the other single-quenching site. These would collapse into fewer than two populations only under special conditions, including cooperative binding of the two OCPs (most likely producing just one population) or if the two non-symmetric sites just happened to produce spectroscopically identical states (two populations).

For more than two sites (for example, for three bound) you would expect to see 1) three bound OCPs, 2) at least one (but likely more than one) quenching level that represents two bound OCPs and 3) at least one quenching level that represents one bound OCP (again, likely more than one). So, for three bound OCPs, we would expect to see at least three or more distinct populations. Again, this possibility cannot be completely eliminated because it is conceivable that strong cooperative binding or similar anomalous factors might prevent some of these combinatorically possible states from being populated. However, in our view the simplest possible explanation for seeing precisely two populations is that two OCPs can bind at symmetric sites.

To illustrate what happens if more than two OCPs are bound, we simulated a three-quencher scenario that includes two symmetric sites (therefore producing some degeneracy and lowering the number of expected states). We selected the quencher strength by requiring that the triply-quenched complex produce the Q_2 photophysical parameters (Supplementary Fig. 11a), and then used this quencher strength to simulate the various possible combinations of two and one quenchers (Supplementary Fig. 11b). As described above, the results predict that this scenario would produce at least 4-5 mutually distinguishable quenched populations.

Supplementary Figures

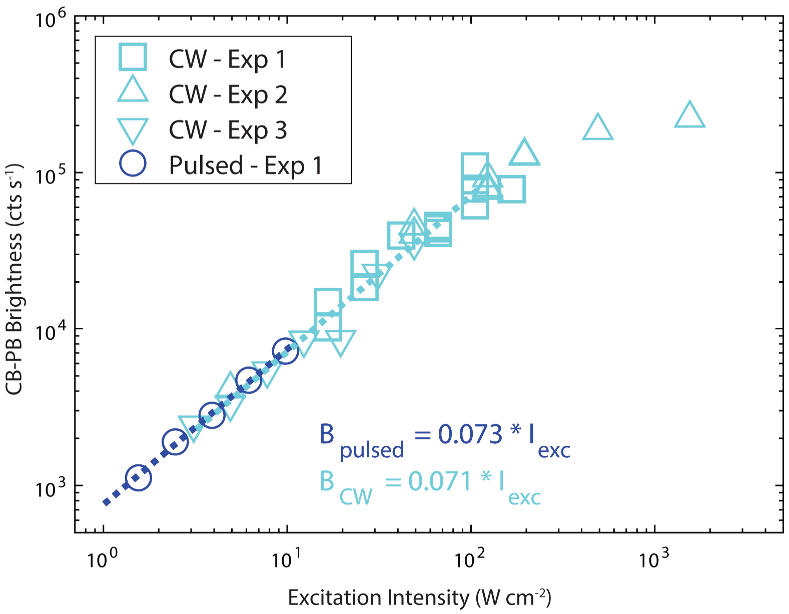

Supplementary Figure 1: Brightness of unquenched CB-PB is linear with excitation intensity
The initial brightness of trapped CB-PB complexes is plotted as a function of excitation intensity in the trap. Nonlinear effects are observed above excitation intensities of ~100 W cm^{-2}, which may be due to either singlet-singlet annihilation, or an extremely brief initial brightness state that is not resolved here. At low excitation intensities on this scale (< 20 W cm^{-2}), little or no photodamage is observed for the unquenched phycobilisome. At higher excitation intensities (> 10 W cm^{-2}), progressive photodamage is apparent in trapped complexes (see Supplementary Figure 2), although the brightness of the initial state continues to be linear with excitation intensity up to ~100 W cm^{-2}, as shown here. Dark blue circles indicate data taken with pulsed excitation (Mira OPO / 80 MHz @ 594 nm), while cyan markers indicate data taken with CW excitation on three different days with re-alignment in between. Least-squares linear fits are shown with dotted lines.

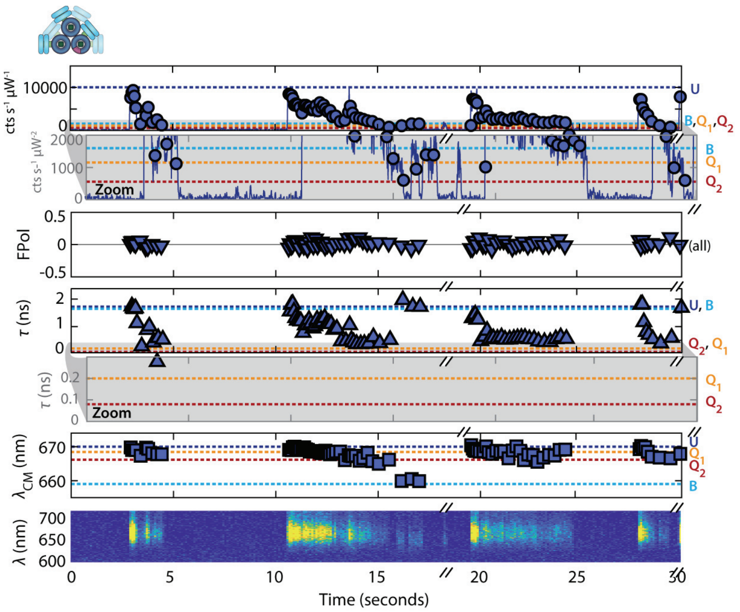

Supplementary Figure 2: Anti-Brownian trapping of single unquenched phycobilisomes under high excitation intensity

Raw trapping data for all parameters (Br, FPol, τ, λ_{CM}, and Em(λ)) for the CB-PB complex in the absence of OCP under high incident excitation intensity (50 W cm^{-2}) shows progressive photodegradation and blinking of the phycobilisome. Data is plotted in either 800-photon groups (markers) or 20-ms bins. Notably, while photodamaged CB-PBs can reach the brightness levels of Q_1 and Q_2, the lifetime remains at or above ~0.3 ns even for large amounts of photodamage. This is clearly distinct from the lifetimes observed for Q_1 and Q_2, τ_{Q1} = 0.21 ns and τ_{Q2} = 0.09 ns.

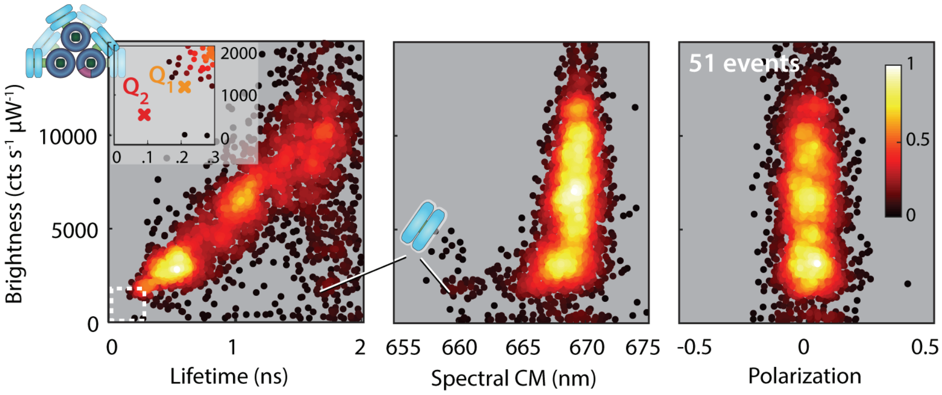

Supplementary Figure 3: Scatter density plots for unquenched phycobilisome at high excitation power

Scatter heatmap of the photophysical states (U_{damage}) observed for the unquenched CB-PB phycobilisome excited at high power (25 W cm^{-2}), shown in Br-τ, Br-λ_{CM}, and Br-FPol projections (left, right, and center, respectively). Each point represents 250 photons, colored according to the local density of points. The location of state B, very rarely observed here, is also indicated for reference. Despite the heterogeneity of photophysical states observed, Q_1 and Q_2 are not observed in this sample (see inset). Colorbar indicates scatter plot coloration according to normalized local density of data points within each panel.

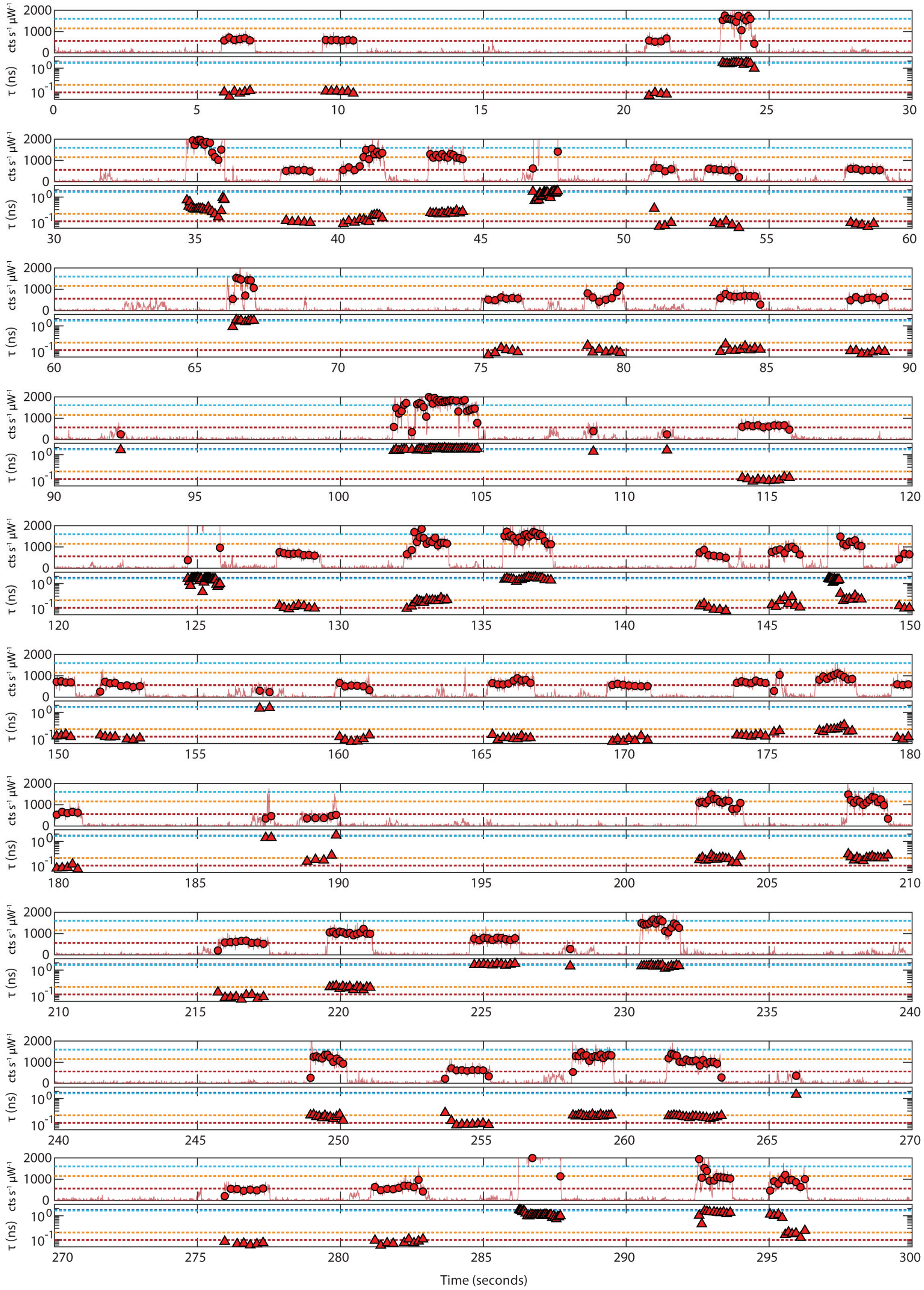
cts s⁻¹ μW⁻¹
τ (ns)
Time (seconds)

Supplementary Figure 4: (Previous page) Extended raw data trace for ABEL trapping of single unquenched phycobilisomes Raw trapping data is shown for Br and τ for OCP-quenched CB-PB complexes (40:1 OCP:CB-PB) over a representative 5-minute data set. Most events contain a single state, and those that do not (e.g.: at 41 sec, at 147 sec, and at 293 sec) may represent replacement events rather than actual transitions among states (see also SI Note S1 and SI Fig. S9). Photophysical parameters within individual states are generally stable over time. Only three trapping events are likely to represent unquenched CB-PB complexes (at 47 sec, 125 sec, and 147 sec), and several free C-PC hexamers are observed (at 24 sec, 67 sec, 103 sec, 137 sec, 232 sec, and 293 sec). States with very dim brightness but long lifetime may represent free trimers or monomers of C-PC or APC (92 sec, 109 sec, 111 sec, 157 sec, 187 sec, and 295 sec).

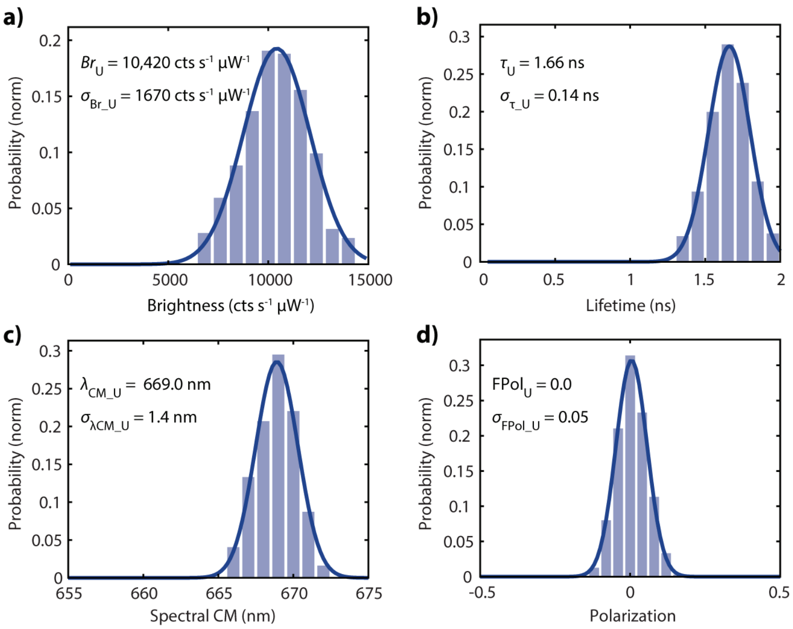

Supplementary Figure 5: Histograms for state U

Gaussian fits for all parameters, a) Br, b) τ, c) λ_{CM}, and d) FPol, for the unquenched CB-PB phycobilisomes (state U), created from the 500-photon groups shown in the scatter plots for Figure 3a. Most likely values and standard deviations in each dimension are also presented in Table 1.

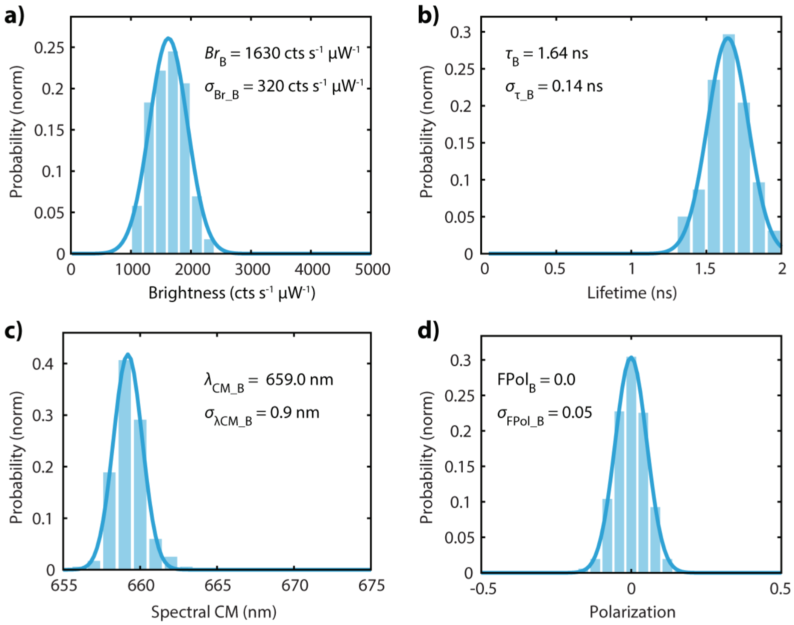

Supplementary Figure 6: Histograms for state B

Gaussian fits for all parameters, a) Br, b) τ, c) λ_{CM}, and d) FPol, for the quenched CB-PB phycobilisomes (state B), created from the 500-photon groups shown in the scatter plots for Figure 3b. Most likely values and standard deviations in each dimension are also presented in Table 1.

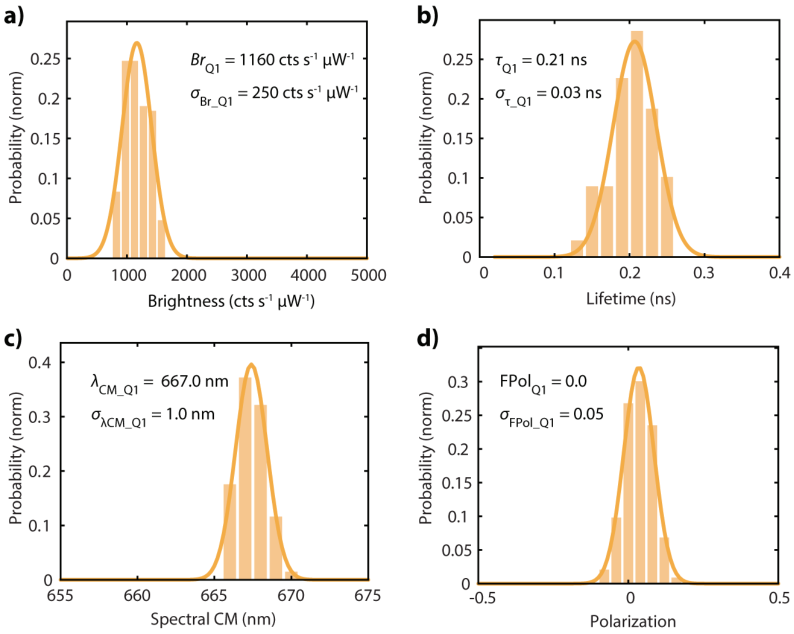

Supplementary Figure 7: Histograms for state Q1

Gaussian fits for all parameters, a) Br, b) τ, c) λ_{CM}, and d) FPol, for the quenched CB-PB phycobilisomes (state Q_1), created from the 500-photon groups shown in the scatter plots for Figure 3b. Most likely values and standard deviations in each dimension are also presented in Table 1.

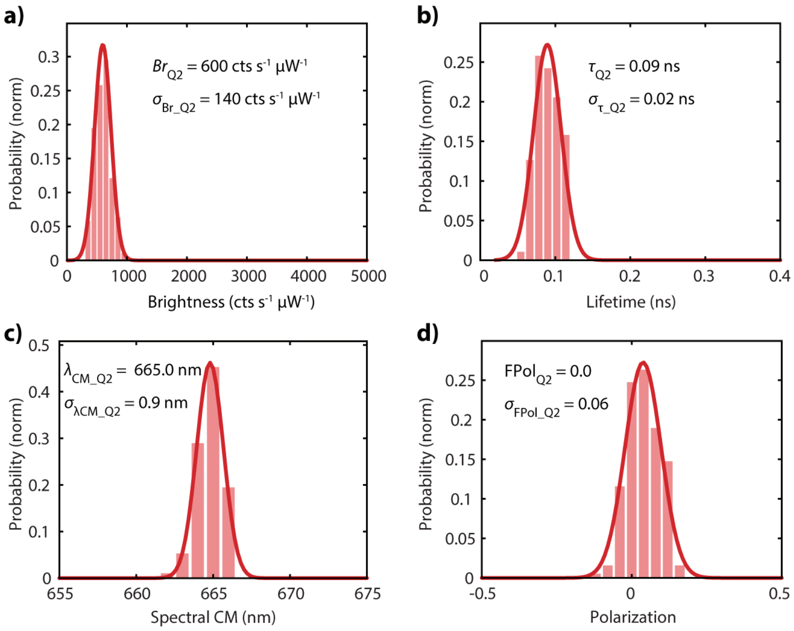

Supplementary Figure 8: Histograms for state Q2

Gaussian fits for all parameters, a) Br, b) τ, c) λ_{CM}, and d) FPol, for the quenched CB-PB phycobilisomes (state Q_2), created from the 500-photon groups shown in the scatter plots for Figure 3b. Most likely values and standard deviations in each dimension are also presented in Table 1.

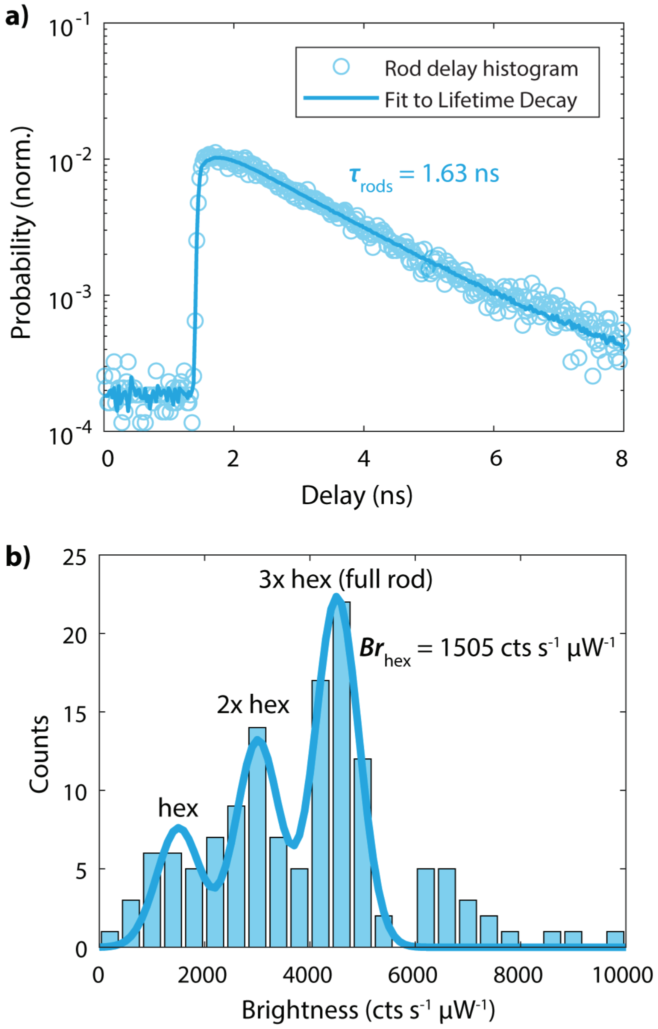

Supplementary Figure 9: Fluorescence lifetime and brightness of C-PC rods in the ABEL trap
C-PC rods from the ΔAB mutant of *Synechocystis* PCC 6803[7] do not include the phycobilisome core. The expected rod structure for this mutant is three hexamers associated face-to-face via the LR33, LR30, and LR9 (rod linker proteins) and CpcG2 rod core linker protein[7,8]. Data was analyzed using the same workflow described in Methods for the CB-PB and CB-PB + OCP data. Some rods dissociate in solution, producing three primary populations: 3-hexamer rods, 2-hexamer rods, and single hexamers. a) Fluorescence lifetime decay histogram for single C-PC rods observed in the ABEL trap. The observed lifetime, 1.63 ns, which is well-fit with a single-exponential decay, closely matches the lifetime observed for state B. b) Histogram and 3-Gaussian fit of brightness data from ABEL trapping of C-PC rods. The above fit was performed under the constraint that $Br_{3Hex} = 3*Br_{1Hex}$ and $Br_{2Hex} = 2*Br_{1Hex}$ to determine the expected brightness and standard deviation for a single C-PC hexamer as $Br_{1Hex} = 1505 \pm 400$ cts s^{-1} μW^{-1}. The brightness of a single C-PC hexamer from this sample is similar to the observed brightness of state B observed in the quenched CB-PB + OCP sample and occasionally observed in the unquenched CB-PB sample (1630 cts s^{-1} μW^{-1}). Further characterization of the CpcG2-PBS mutant will be reported elsewhere[8].

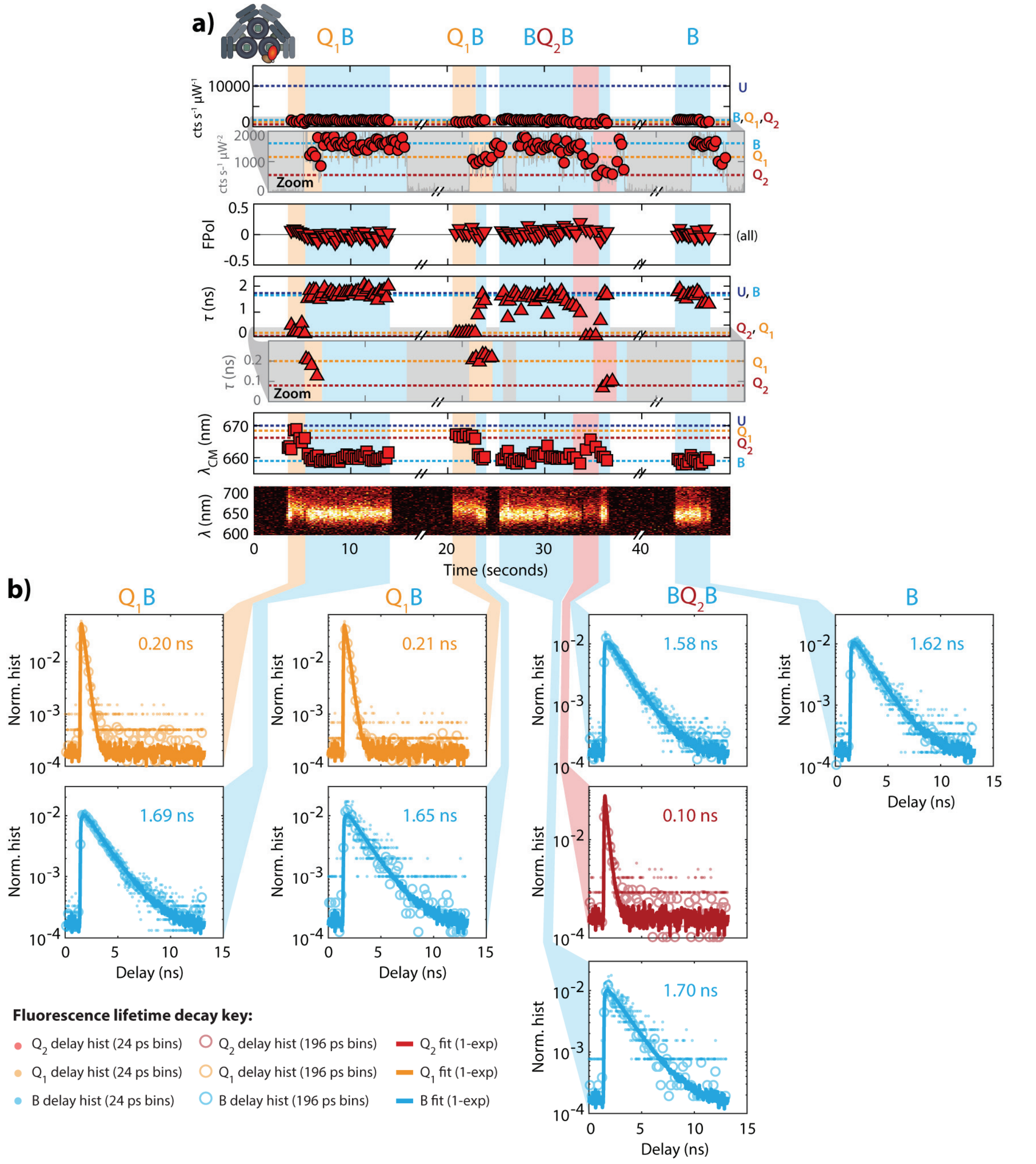

Supplementary Figure 10: The identity of state B and apparent transitions during trapping
a) Raw trapping data for all parameters (Br, FPol, τ, λ_{CM}, and Em(λ)) for the CB-PB + OCP quenched complex at relatively high sample concentration (~30 pM) and relatively high excitation (power: 1 μW, intensity: 30 W cm^{-2}) show apparent transitions among states, particularly to and from state B. However, these changes are likely due to replacement events, where either a hexamer decouples from the CB-PB and becomes the sole trapped object, or when another object enters the trap and becomes the trapped object. b) The lifetime decays and fits for each state within each event are all well-fit by single-exponential models, with fitted lifetimes that closely match the assigned states. Notably, there is no evidence of a short lifetime component in any of the state B decays, which indicates that the trapped object does not include a quenched CB-PB phycobilisome.

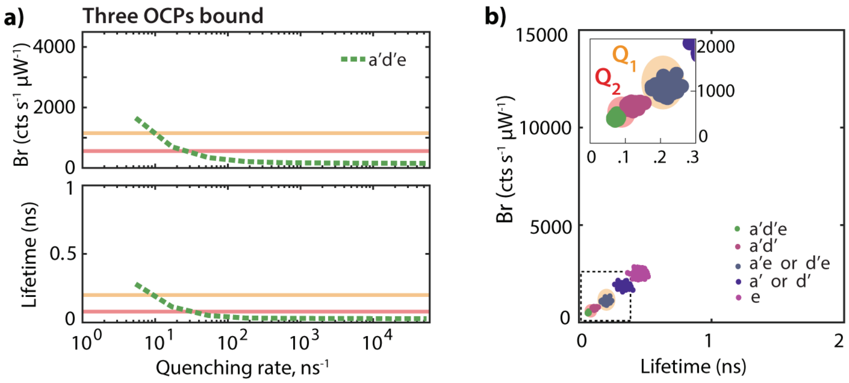

Supplementary Figure 11: Compartmental model of OCP-quenched CB-PB phycobilisome for three quenchers at a', d', and e. a) Brightness and fluorescence lifetime of the CB-PB phycobilisome for three bound quenchers located at sites *a*'*d*'*e*, plotted for increasing quencher strength. This panel illustrates that a quenching strength of 33 ns^{-1} at all three sites predicts that the triply-quenched complex falls on the experimentally measured Q_2 state. Horizontal lines show the experimentally measured parameters for Q_1 (orange) and Q_2 (red). b) Using this value for the OCP quenching rate (33 ns^{-1}) for all three sites, model results in brightness and lifetime are shown for five combinatoric conditions: (1) Three bound quenchers, *a*'*d*'*e*, shown in green. (2) Two bound quenchers at *a*'*d*', shown in dark red. (3) Two bound quenchers at *a*'*e* or *d*'*e*, which happen to produce nearly indistinguishable photophysical states, shown in gray-blue, (4) a single bound quencher at *a*' or *d*', which are symmetric and therefore produce degenerate photophysical states, shown in purple, and (5) a single bound quencher at *e*, shown in magenta. As described above in SI Note 4, the results predict that this scenario would produce 4-5 distinct quenched populations, rather than the two that were observed.

Supplementary Tables

Current Compartment (columns) / Transfer Compartment (rows)

Transfer Compartment	CPC Top Left	CPC Top Right	CPC Bottom Left One	CPC Bottom Right One	CPC Bottom Left Two	CPC Bottom Right Two	APC 660 Top Left	APC 660 Top Right	APC 660 Bottom Left One	APC 660 Bottom Right One	APC 680 Right	APC 660 Bottom Left Two	APC 660 Bottom Right Two	APC 680 Left	OCP
CPC Top Left							5								
CPC Top Right								5							
CPC Bottom Left One									5						
CPC Bottom Right One										5					
CPC Bottom Left Two												5			
CPC Bottom Right Two													5		
APC 660 Top Left	37							92	8			8			
APC 660 Top Right		37					92			8			8		
APC 660 Bottom Left One			27				6			69		6		70	
APC 660 Bottom Right One				37				8	92				8		
APC 680 Right													234		
APC 660 Bottom Left Two					37		8		8				92		
APC 660 Bottom Right Two						27		6		6	70	69			
APC 680 Left									234						
OCP							Variable Strength								
Fluorescence Emission	0.6	0.6	0.6	0.6	0.6	0.6	0.6	0.6	0.6	0.6	0.6	0.6	0.6	0.6	

0.6	Very Slow	5-8	Slow	27-37	Medium	69-92	Fast	234	Very Fast

Supplementary Table 1: Connectivity of compartmental model

Rate matrix showing the connectivity of the compartmental model and the associated rates of forward and backward transfer for each connected pair of compartments. All rates are in ns^{-1}. Quenching via OCP during different simulations is turned on (given a baseline rate of 54 ns^{-1}) or off (given a rate of 0 ns^{-1}) to test different quenching sites and different combinations of sites. Which compartments have an active quencher for a given simulation is stated in the main text.

Compartment	Normalized Probability of Absorption
CPC Top Left	0.11
CPC Top Right	0.11
CPC Bottom Left One	0.11
CPC Bottom Right One	0.11
CPC Bottom Left Two	0.11
CPC Bottom Right Two	0.11
APC 660 Top Left	0.051
APC 660 Top Right	0.051
APC 660 Bottom Left One	0.051
APC 660 Bottom Right one	0.051
APC660 Bottom Left Two	0.051
APC660 Bottom Right Two	0.051
APC 680 Left	0.019
APC 680 Right	0.019

Supplementary Table 2: Compartmental model absorption probabilities
Probability of populating a compartment for Monte Carlo simulations. Probability is based on the normalized molar extinction coefficients of C-PC and APC at 590 nm.

Supplementary References

1. Yang, H.; Moerner, W. Precise Measurement of Single-Molecule Rotational Diffusivity in Solution. *Biophys. J.* **114**, 170a (2018).

2. Wang, Q.; Moerner, W. E. Single-molecule motions enable direct visualization of biomolecular interactions in solution. *Nat. Methods***.** **11**, 555-558 (2014).

3. Cohen, A. E.; Moerner, W. E. Principal-components analysis of shape fluctuations of single DNA molecules. *Proc. Natl. Acad. Sci. U. S. A.* **104**, 12622-12627 (2007).

4. Schlau-Cohen, G. S.; Wang, Q.; Southall, J.; Cogdell, R. J.; Moerner, W. E. Single-molecule spectroscopy reveals photosynthetic LH2 complexes switch between emissive states. *Proc. Natl. Acad. Sci. U. S. A.* **110**, 10899-10903 (2013).

5. Wang, Q.; Moerner, W. E. Dissecting pigment architecture of individual photosynthetic antenna complexes in solution. *Proc. Natl. Acad. Sci. U. S. A* **112**, 13880-13885 (2015).

6. Squires, A. H.; Moerner, W. E. Direct single-molecule measurements of phycocyanobilin photophysics in monomeric C-phycocyanin. *Proc. Natl. Acad. Sci. U. S. A.* **114**, 9779-9784 (2017).

7. Ajlani, G.; Vernotte, C.; DiMagno, L.; Haselkorn, R. Phycobilisome core mutants of Synechocystis PCC 6803. *BBA-Bioenergetics,* **1231,** 189-196 (1995).

8. Liu, H., Weisz, D.A., Zhang, M., Zhang, H., Cheng, M., Gerstenecker, G.S., Pakrasi, H.B., Gross, M.L., Blankenship, R.E. Cross-linking and protein structure prediction reveal close association of FNR with CpcB in *Synechocystis sp.* PCC 6803 (submitted).
